# Oxidation of Monolignols by Members of the Berberine Bridge Enzyme Family Suggests a Role in Plant Cell Wall Metabolism*[Fn FN2]

**DOI:** 10.1074/jbc.M115.659631

**Published:** 2015-06-02

**Authors:** Bastian Daniel, Tea Pavkov-Keller, Barbara Steiner, Andela Dordic, Alexander Gutmann, Bernd Nidetzky, Christoph W. Sensen, Eric van der Graaff, Silvia Wallner, Karl Gruber, Peter Macheroux

**Affiliations:** From the Institutes of ‡ Biochemistry,; ‖ Biotechnology and Biochemical Engineering, and; ** Molecular Biotechnology, Graz University of Technology, 8010 Graz, Austria,; the § Institute of Molecular Biosciences, University of Graz, 8010 Graz, Austria,; the ¶ ACIB GmbH, 8010 Graz, Austria, and; the ‡‡ Section for Crop Sciences, Copenhagen University, 2630 Copenhagen, Denmark

**Keywords:** Arabidopsis thaliana, enzyme catalysis, flavin adenine dinucleotide (FAD), flavoprotein, plant biochemistry

## Abstract

Plant genomes contain a large number of genes encoding for berberine bridge enzyme (BBE)-like enzymes. Despite the widespread occurrence and abundance of this protein family in the plant kingdom, the biochemical function remains largely unexplored. In this study, we have expressed two members of the BBE-like enzyme family from *Arabidopsis thaliana* in the host organism *Komagataella pastoris*. The two proteins, termed *At*BBE-like 13 and *At*BBE-like 15, were purified, and their catalytic properties were determined. In addition, *At*BBE-like 15 was crystallized and structurally characterized by x-ray crystallography. Here, we show that the enzymes catalyze the oxidation of aromatic allylic alcohols, such as coumaryl, sinapyl, and coniferyl alcohol, to the corresponding aldehydes and that *At*BBE-like 15 adopts the same fold as vanillyl alcohol oxidase as reported previously for berberine bridge enzyme and other FAD-dependent oxidoreductases. Further analysis of the substrate range identified coniferin, the glycosylated storage form of coniferyl alcohol, as a substrate of the enzymes, whereas other glycosylated monolignols were rather poor substrates. A detailed analysis of the motifs present in the active sites of the BBE-like enzymes in *A. thaliana* suggested that 14 out of 28 members of the family might catalyze similar reactions. Based on these findings, we propose a novel role of BBE-like enzymes in monolignol metabolism that was previously not recognized for this enzyme family.

## Teaser

**Background:** Berberine bridge enzyme-like proteins are a multigene family in plants.

**Results:** Members of the berberine bridge enzyme-like family were identified as monolignol oxidoreductases.

**Conclusion:** Berberine bridge enzyme-like enzymes play a role in monolignol metabolism and lignin formation.

**Significance:** Our results indicate a novel and unexpected role of berberine bridge enzyme-like enzymes in plant biochemistry and physiology.

## Introduction

Flavoproteins are a large and diverse protein family employing the FAD cofactor for catalysis. Among them the berberine bridge enzyme (BBE)^2^-like proteins (pfam 08031) can be set apart due to their unusual bicovalent attachment of the FAD cofactor. The namesake of this protein family is BBE from *Eschscholzia californica* (California poppy) that catalyzes the formation of the so-called “berberine bridge” by oxidation of the *N*-methyl group of (*S*)-reticuline yielding (*S*)-scoulerine (1). This step constitutes a branch point in the biosynthesis of numerous isoquinoline alkaloids (2, 3). In recent years, a large number of genes encoding BBE-like enzymes have been identified in plants and bacteria in the course of genome sequencing efforts. The number of BBE-like genes in individual plant species varies considerably from a single gene in the moss *Physcomitrella patens* to 64 in the western poplar (*Populus trichocarpa*) (4). In the model plant *Arabidopsis thaliana*, 28 BBE-like genes were identified (termed *At*BBE-like 1–28 (4)). However, the role of these BBE homologs in *A. thaliana* and most of the other plants is unknown, as most do not synthesize alkaloids of the benzylisoquinoline family. Examination of microarray data (5) revealed the expression of *At*BBE-like genes during certain developmental stages, such as root elongation and maturation, and proliferation as well as embryonal development (Table 1). Similarly, osmotic stress and pathogen attack were shown to cause up-regulation of *AtBBE*-likes by up to 400-fold. This up-regulation was also seen in other plants, such as citrus fruit and poplar (6, 7). The role in pathogen defense appears to be related to a carbohydrate oxidase activity that was reported previously for tobacco (nectarin V), sunflower, and lettuce (8, 9). Similar oxidation reactions occur in the biosynthesis of antibiotics generated in various bacterial species, such as *Streptomyces* (10–12). However, the role of BBE homologs in plant development remains enigmatic.

**TABLE 1:** **Expression of *At*BBE-homologs *in planta*** Data were retrieved from the *Arabidopsis* eFP browser.

Subgroup	Name	Most significant expression value/up-regulation	Second significant expression value/up-regulation	Localization, MS/MS	Locus
1	*At*BBE-like 18	822, seed linear cotyledon		Unknown	AT4G20820.1
1	*At*BBE-like 27	1117, root		Unknown	AT5G44410.1
1	*At*BBE-like 28	6000, shift dark to light	927, quiescent center root	Extracellular	AT5G44440.1
2	*At*BBE-like 3	2457/438-fold osmotic stress seedling	2318/457-fold, infection *P. infestans*	Endoplasmic reticulum	AT1G26380.1
2	*At*BBE-like 4	2070/78-fold infection, *P. syringae*	391, root regeneration	Unknown	AT1G26390.1
2	*At*BBE-like 5	665, cotyledons	475, cotyledon greens	Unknown	AT1G26400.1
2	*At*BBE-like 6	604/42-fold, osmotic stress	278/36-fold infection, *P. syringae*	Unknown	AT1G26410.1
2	*At*BBE-like 7	1562/412-fold, osmotic stress	800/163-fold, mock infection	Unknown	AT1G26420.1
2	*At*BBE-like 17	7772, micropylar endosperm		Unknown	AT4G20800.1
3	*At*BBE-like 10	4899, lateral root cap	912/57-fold, mock infection	Unknown	AT1G30720.1
3	*At*BBE-like 11	4988, root epidermis	762/10-fold, infection *P. syringae*	Extracellular	AT1G30730.1
4	*At*BBE-like 9	7582, mature pollen		Unknown	AT1G30710.1
4	*At*BBE-like 14	50, seed		Unknown	AT1G34575.1
4	*At*BBE-like 16	3682, root maturation zone	1537, stigma and ovaries	Unknown	AT2G34810.1
5	*At*BBE-like 1	11,626, pollen	5509, mature pollen	Plasma membrane, Golgi	AT1G01980.1
5	*At*BBE-like 2	5088, pollen	2000, pollen tubes	Unknown	AT1G11770.1
5	*At*BBE-like 8	2557, root maturation zone	1157/246-fold infection, *P. syringae*	Unknown	AT1G30700.1
5	*At*BBE-like 12	642, endosperm		Unknown	AT1G30740.1
5	*At*BBE-like 20	4000, root elongation zone	1394/10-fold infection *P. syringae*	Extracellular, plasma membrane	AT4G20830.2
5	*At*BBE-like 21	973, root elongation zone		Plasma membrane	AT4G20840.1
6	*At*BBE-like 13	2144, lateral root initiation	1594, xylem root maturation zone	Unknown	AT1G30760.1
6	*At*BBE-like 15	4927, xylem root	439, seed stage 8 curled	Extracellular, plasma membrane	AT2G34790.1
6	*At*BBE-like 24	32,075, lateral root cap	1412, cotyledon	Extracellular	AT5G44380.1
6	*At*BBE-like 25	491, procambium root	164/5-fold infection *P. syringae*	Extracellular	AT5G44390.1
6	*At*BBE-like 26	604/5-fold osmotic stress root	250/3-fold infection *P. syringae*	Extracellular	AT5G44400.1
7	*At*BBE-like 22	1641, lateral root initiation	1427/13-fold infection *P. syringae*	Plasma membrane, cytosol	AT4G20860.1
7	*At*BBE-like 23	824, root	649, seed cotyledon heart stage	Unknown	AT5G44360.1

For this study, we have chosen *At*BBE-like 15 (At2g34790) for further studies into the role of the BBE-like enzyme family in plants because gene disruption resulted in defects in female gametophyte development (*i.e.* unfused polar nuclei and endosperm development arrest) (13). Furthermore, mass spectrometric analysis has shown that *At*BBE-like 15 is located in the cell wall and thus might participate in as yet undefined reactions relevant for the formation of the cell wall (14). In addition, we have selected *At*BBE-like 13 (At1g30760) for further analysis due to its close sequence relationship of 81% identity to *At*BBE-like 15.

Structural analysis of recombinant *At*BBE-like 15 by x-ray crystallography confirmed a topology similar to that of berberine bridge enzyme from *E. californica* (*Ec*BBE) and Δ1-tetrahydrocannabinolic acid synthase (THCA synthase) from *Cannabis sativa* (15, 16). However, composition and architecture of the active site of *At*BBE-like 15 indicated distinct catalytic properties. To identify potential substrates, we screened a small compound library with regard to its effect on the protein's thermal stability. This approach produced several hits and allowed the identification of cinnamyl alcohol as a lead structure. Further analysis showed that the *p*-hydroxylated derivatives of cinnamyl alcohol, *i.e.* the monolignols *p*-coumaryl-, coniferyl-, and sinapyl alcohol are rapidly oxidized to their corresponding aldehydes. Furthermore, the β-*O*-glycosylated form of coniferyl alcohol (coniferin) is also accepted as a substrate. Because monolignols and their β-glycosylated derivatives (coniferin, syringin, and *p*-coumaryl-β-glycoside) are important building blocks and monolignol storage forms, respectively, the catalytic reactions performed by *At*BBE-like 13 and *At*BBE-like 15 constitute a novel link between the phenylpropanoid pathway and the formation of plant polymerization products, such as lignin and suberin. The characteristic active site found in *At*BBE-like 13 and *At*BBE-like 15 is conserved in the majority of BBE-like enzymes in *A. thaliana* (14 of 28) suggesting that they either exhibit different substrate preferences or have distinct spatial (*e.g.* different plant tissues) or temporal (*e.g.* in response to pathogens or herbivores) functions *in planta*.

## Experimental Procedures

### 

#### 

##### Chemicals

All chemicals were from Sigma and were of the highest grade commercially available. Restriction enzymes were obtained from Fermentas (Waltham, MA). Nickel-Sepharose 6 Fast Flow column material was from GE Healthcare (Little Chalfont, UK). Synthetic genes coding for *At*BBE-like 15 and *At*BBE-like 13 were obtained from Life Technologies, Inc., with codon usage optimized for *Komagataella pastoris*.

##### Molecular Cloning

The proteins were expressed using *Komagataella pastoris* as expression host, according to the EasySelect^TM^ *Pichia* expression kit provided by Invitrogen. The genes were adapted to the *K. pastoris* codon usage, and a C-terminal His tag was added. SignalP was used to identify the native signal sequence of 30 and 27 amino acids for *At*BBE-like 13 and *At*BBE-like 15, respectively (17). The genes lacking the signal sequence were cloned into pPICZα vector® (Invitrogen) using standard techniques. *K. pastoris* strain KM71H was transformed with the pPICK-PDI vector harboring the gene for the protein-disulfide isomerase from *Saccharomyces cerevisiae*. The modified KM71H strain was transformed with the linearized [pPICZα-*At*BBE-like 13] or [pPICZα-*At*BBE-like 15] construct using electroporation. Optimal expression strains were identified using the method proposed by Weis *et al.* (18).

##### Protein Expression and Purification

Expression was carried out using a BBI CT5-2 fermenter (Sartorius, Göttingen, Germany) using a basal salt minimal medium as described by Schrittwieser *et al.* (19). After 96 h of methanol induction, the pH was set to 8.0 with sodium hydroxide, and imidazole was added to a final concentration of 10 mm. The cells were removed by centrifugation at 4000 rpm at 4 °C for 30 min. The supernatant was incubated with 50 ml of nickel-Sepharose 6 Fast Flow material at 4 °C for 45 min. Then the affinity material was packed into a column and washed with 5 column volumes of 50 mm phosphate buffer, pH 8.0, containing 150 mm NaCl and 20 mm imidazole. The protein was eluted using 50 mm phosphate buffer, pH 8.0, containing 150 mm NaCl and 150 mm imidazole. Fractions containing *At*BBE-like 15 were concentrated using Amicon Ultracentrifugal filter units and subsequently loaded on a Superdex 200 gel filtration column using an Äkta system and separated using 50 mm Tris buffer, pH 8.0, containing 150 mm sodium chloride. The purity of purified protein was monitored by SDS-PAGE. The final yield from 3.5 liters of fermentation supernatant was 520 mg of purified protein. The large scale expression of *At*BBE-like 13 was conducted as described for *At*BBE-like15. In contrast to *At*BBE-like 15, *At*BBE-like 13 was not secreted, and hence the protein was isolated from cells and not the cultivation medium. Briefly, the cell pellet was harvested and redissolved (1:3 w/v) in cell lysis buffer (50 mm NaH_2_PO_4_, 300 mm NaCl, 20 mm imidazole, pH 8.0), disrupted by zirconia/silica beads using a Merckenschlager homogenizer. The lysate was separated from the cell debris by centrifugation (18000 rpm, 30 min, 4 °C), filtered, and incubated with nickel-Sepharose 6 Fast Flow material (GE Healthcare). The column was packed with the Sepharose material, and after extensive washing (50 mm NaH_2_PO_4_, 300 mm NaCl, 50 mm imidazole, pH 8.0), the protein was eluted with elution buffer containing 500 mm imidazole. Subsequent purification was performed as described for *At*BBE-like 15.

##### Site-directed Mutagenesis, Generation of the L182V Variant

Site-directed mutagenesis was performed according to the instructions of the QuikChange XL kit (Stratagene, La Jolla, CA) for site-directed mutagenesis. The pPICZα-*At*BBE-like 15 vector was used as template for the polymerase chain reaction. The introduction of the desired codon causing a change of leucine in position 182 to valine was verified by sequencing. Transformation of the gene was performed as described before. Expression of the mutated gene and purification of the *At*BBE-like 15 L182V variant was performed as described for wild-type *At*BBE-like 15.

##### Crystallization

Initial crystal screening of *At*BBE-like 15(27–532) was performed with an Oryx8 robot (Douglas Instruments, Berkshire, UK) using commercially available Index (Hampton Research, Aliso Viejo, CA) and JCSG+ (Molecular Dimensions, Suffolk, UK) screens. Screening was performed in Swissci triple well plates (Molecular Dimensions) using the sitting drop method with a reservoir volume of 33 μl. Drops of 1 μl were pipetted in a 1:1 ratio of protein and reservoir solution with two different protein stock solutions, 22 and 50 mg/ml in 50 mm Tris buffer, pH 8.0, containing 150 mm sodium chloride, respectively. Plates were sealed with the thermal seal A sealing films (Sigma) and were incubated at 289 K. First crystals appeared after 1 week in JCSG+ screen condition 1–14 (0.2 m sodium thiocyanate and 20% w/v PEG 3350) with the higher protein concentration. Crystals from this drop were pipetted out and crushed in 100 μl of the original 1–14 solution and used as a seeding stock. Cross-seeding setups (20) were performed in the same manner as initial screening with drops consisting of 0.5 μl of protein stock solution (37 mg/ml), 0.2 μl of the seeding stock, and 0.5 μl of the JCSG+ screen solutions from the reservoir. The best diffracting crystals appeared after 4 and a half weeks in a serial dilution setup done manually in Crystal Clear duo plates for sitting drop (Douglas Instruments). The 1.2-μl drops consisted of 0.5 μl of protein stock solution (36 mg/ml), 0.2 μl of the seeding stock, and 0.5 μl of the JCSG+ screen condition 2-33 (0.1 m potassium thiocyanate and 30% w/v PEG 2000 MME) from the reservoir.

##### Data Collection and Processing

X-ray diffraction data were collected at 100 K at Elettra (Trieste) without additional cryoprotectant. Data processing was performed with the XDS program package (21). Unit cell parameters and assigned space groups as well as data statistics are shown in Table 3. The solvent content was estimated based on the calculated Matthews coefficient (22). Molecular replacement was performed with Phaser (23) using the structure of *Ec*BBE as the search template. Structure refinement was done with Phenix (24) followed by manual inspection and model rebuilding in Coot (25). The program Readyset! (included in Phenix) was used to generate restraints for metal coordination and the PEG fragment included in the refinement. In total, three sodium and one potassium ion were found to bind to the protein surface without structural impact. The TLSMD server was used to generate partitioning groups (three for chain A and four for chain B) to improve refinement (26). Several refinement cycles were done until all visible residues were assigned, and no significant changes in *R* and *R*free were observed. Structure validation was performed using MolProbity (27).

Chain A of the model consists of residues Ser-27 to Gly-532. Chain B starts with residue Gln-30 and extends to Gly-532. Some residues in the N-terminal region could not be modeled into the electron density most likely due to their high flexibility (Val-43 and Ser-44 in chain A as well Gln-40, Ser-41, and Asp-42 in chain B). In addition, no clear electron density was visible for a loop region (residues 301–305) in both chains.

##### Thermofluor Experiments

Thermofluor experiments were performed using a CFX Connect real time PCR system (Bio-Rad). The experiments were performed using Sypro® Orange as fluorescent dye in 50 mm MES buffer, pH 7.0. Stock solutions of all substrates were prepared in water with a concentration of 10 mg/ml. For substrates with a lower solubility, a saturated solution was used. The total volume in each well was 25 μl with a protein concentration of 0.4 and 2 mg/ml substrate. The starting temperature of 20 °C was kept for 5 min and then the temperature was increased at a rate of 0.5 °C/min to 95 °C. Melting temperatures were determined using the program Bio-Rad CFX Manager 3.0.

##### Rapid Reaction Kinetics Using Stopped-flow Spectrophotometry

Reductive and oxidative half-reactions were determined with a stopped-flow device (SF-61DX2, TgK Scientific, UK) at 25 °C under anoxic conditions in a glove box (Belle Technology, Weymouth, UK). Oxygen was removed from the sample by flushing with nitrogen and subsequent incubation of the samples in the glove box for 1 h. Spectral changes of the flavin cofactor were followed at a wavelength of 450 nm using a KinetaScanT diode array detector (MG-6560, Hi-Tech) employing the Kinetic Studio software (TgK Scientific, UK). Reductive half-reactions were assayed by mixing 60 μm *At*BBE-like 15 or 13 in 50 mm potassium phosphate buffer, pH 7.0, with various substrate concentrations. The observed rate constants at different substrate concentrations (*k*_obs_) were determined using the Kinetic Studio software by fitting the data to an exponential function. If suitable, the dissociation constant was determined performing a sigmoidal fit of the observed rate constants employing Origin 7 (OriginLab Corp., Northampton). The oxidative half-reaction was determined by mixing oxygen free photo-reduced 60 μm *At*BBE-like 15, *At*BBE-like 15 L182V or *At*BBE-like 13 with air-saturated buffer. Photoreduction was carried out according to Massey and Hemmerich (28).

##### Product Identification

Products resulting from the conversion of monolignols and their glycosides were separated and identified using HPLC. Reactions were performed in 1.5-ml reaction vials in 50 mm potassium phosphate buffer, pH 7.0, at 30 °C and 700 rpm. Substrates were used at a concentration of 2.5 mm and 30 μg/ml *At*BBE-like 15. After 24 h, the reaction was quenched by mixing a 500-μl sample with 500 μl of methanol. Samples were spun for 10 min at 9400 × *g* in a bench-top centrifuge before the clear supernatant was applied to the HPLC. The products were identified by retention time and by comparing their UV absorption spectra with authentic standards. HPLC analyses were done using a Dionex UltiMate 3000 HPLC (Thermo Fisher Scientific, Waltham, MA) equipped with an Atlantis® dC18 5 μm (4.6 × 250 mm) column. Separation of all compounds was achieved using a linear gradient with water with 0.1% TFA as solvent A and acetonitrile with 0.1 TFA as solvent B and a flow rate of 0.5 ml/min. Separations were started with a mobile phase of 80% solvent A and 20% solvent B. The concentration of solvent B was increased to 50% within 20 min followed by a steep ramp to 100% solvent B in 10 min. At the end of the protocol, the concentration of solvent B was again decreased to 20% over 5 min. Retention times of authentic standard compounds were determined using the described protocol. Under these experimental conditions, the following retention times were observed: coniferyl alcohol, 16.06 min; coniferyl aldehyde, 21.39 min; ferulic acid, 18.27 min; coumaryl alcohol, 15.52 min; coumaryl aldehyde, 21.07 min; *p*-coumaric acid, 17.67 min; sinapyl alcohol, 15.40 min; sinapyl aldehyde, 21.01 min, and sinapic acid, 17.76 min.

##### Synthesis of Monolignol Glycosides

A glucosyltransferase from apple (UGT71A15, *Malus* x *domestica*) was used for the synthesis of 4-*O*-β-d-glucopyranosides of the monolignols (29). Heterologous expression in *Escherichia coli* and purification of the glycosyltransferase will be published elsewhere. Briefly, glucosylation of the aglyca (5 mm) from 7.5 mm uridine UDP-glucose was performed in 50 mm Tris/Cl buffer, pH 7.5, containing 50 mm MgCl_2_, 0.13% BSA, and 10% DMSO in the presence of 6 μm UGT71A15.

##### Phylogenetic Tree Construction

M-Coffee was used to create a multiple sequence alignment, including all 28 *At*BBE-like family members (30). The alignment was edited by hand using Jalview (31). The PHYLIP package (PHYLIP 3.69) was used to create a bootstrapped phylogenetic tree using the programs SEQBOOT, PROTDIST, FITCH, and CONSENSE (32). We created 1000 Jackknife sub-alignments with SEQBOOT, which were subsequently subjected to a bootstrapped protein-distance analysis. We chose *Ec*BBE as the outgroup sequence. The tree shown in supplemental Fig. S1 was visualized using FigTree (Tree Figure Drawing Tool, version 1.4.0 by Andrew Rambaut). The accession codes for the sequences used for the analysis are given in the legend of supplemental Fig. S1.

##### Docking

Docking of coniferyl alcohol was performed using AutoDock Vina using the default docking parameters (33, 34). Point charges were assigned according to the AMBER03 force field (35). The setup of the receptor was done with the YASARA molecular modeling program (36). Twenty five Vina docking runs were performed, and after clustering all runs, four distinct complex conformations with a binding energy of −6.4, −6.1, −5.0, and −4.6 kJ mol^−1^ were found. The complex with a binding energy of −6.4 kJ mol^−1^ and a conformation that is in agreement with the catalytic activity found for AtBBE-like 15 is shown in Fig. 3. 8% of the docks cluster in this top pose.

## Results

### 

#### 

##### Enzymatic Properties and Identification of Substrates of AtBBE-like 13 and AtBBE-like 15

**FIGURE 1.**
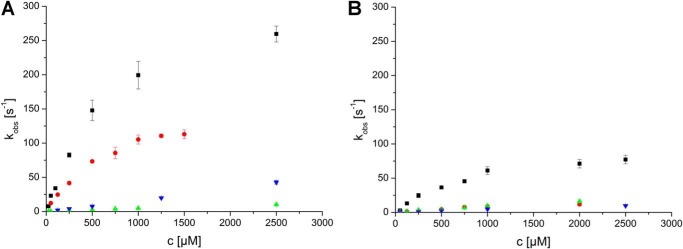
**Rate constants observed for the reductive half-reaction of *At*BBE-like 15 (*A*) and *At*BBE-like 13 (*B*).** A stopped-flow device was used to determine the rate constants of the reductive half-reaction of *At*BBE-like 15 and 13 with *p*-coumaryl alcohol (*solid square*), cinnamyl alcohol (*green triangle*), coniferyl alcohol β-glycoside (*orange circle*), and sinapyl alcohol β-glycoside (*inverted blue triangle*). The course of the reaction was determined by following the absorption of the oxidized flavin at 450 nm. As the reactions follow pseudo-first order kinetics, rates were calculated from a monoexponential fit.

*At*BBE-like 13 and *At*BBE-like 15 were expressed in *K. pastoris* and purified from the culture medium by nickel-Sepharose affinity chromatography and subsequent gel filtration yielding ∼10 and 150 mg of protein from 1 liter of fermentation culture, respectively. To identify potential substrates, we inspected the chemical nature of substrates recently identified for vanillyl alcohol oxidase (VAO) superfamily members. This revealed that oxidation of primary and secondary alcohol groups is a prevailing feature in all of these reactions (4). In view of this recurring motif, we have assembled a library comprising predominantly compounds with one or more hydroxyl groups (see supplemental Table S1). Because strong protein-ligand interactions are known to increase the thermal stability of proteins, we first screened our library with regard to an increase in the melting temperature of *At*BBE-like 15 using the ThermoFluor method (37). This approach produced a number of promising hits, yielding a substantial increase of the melting temperature (up to 17 °C, for a complete list see supplemental Table S1). The compounds with the strongest effect on the thermal stability were tested as substrates revealing that cinnamyl alcohol was oxidized to cinnamyl aldehyde by *At*BBE-like 15. Using cinnamyl alcohol as a lead structure, we tested several other naturally occurring aromatic alcohols as putative substrates and found that *At*BBE-like 15 oxidizes the monolignols *p*-coumaryl-, coniferyl-, and sinapyl alcohol to their corresponding aldehydes. To gain more detailed information on substrate specificity and kinetic parameters, reductive half-reactions were studied using stopped-flow spectrophotometry (a summary of kinetic parameters is given in Table 2). Reduction of *At*BBE-like 15 and 13, using either coniferyl or sinapyl alcohol, was very fast and essentially complete within the dead time of the instrument (∼5 ms) indicating that the rate of reduction exceeds 500 s^−1^. In the case of *p*-coumaryl alcohol, the rate of reduction could be analyzed as a function of substrate concentration, yielding a dissociation constant of 700 μm and a limiting rate of 332 s^−1^. Because monosaccharides, such as d-glucose, had such a profound effect on the protein's thermal stability, we also analyzed the β-*O*-glycosylated derivatives of the monolignols and found that coniferin was accepted as a substrate exhibiting a dissociation constant of 660 μm and a limiting rate of reduction of 171 s^−1^. However, the β-*O*-glycosylated derivatives of *p*-coumaryl and sinapyl alcohol (syringin) were relatively poor substrates of the enzyme. An equivalent set of experiments was conducted with *At*BBE-like 13 demonstrating similar catalytic properties (summarized in Table 2). The observed rate constants for selected substrates determined for *At*BBE-like 15 and 13 are shown in Fig. 1, *A* and *B*. Thus, we propose that *At*BBE-like 13 and *At*BBE-like 15 are in fact monolignol oxidoreductases.

**TABLE 2:**
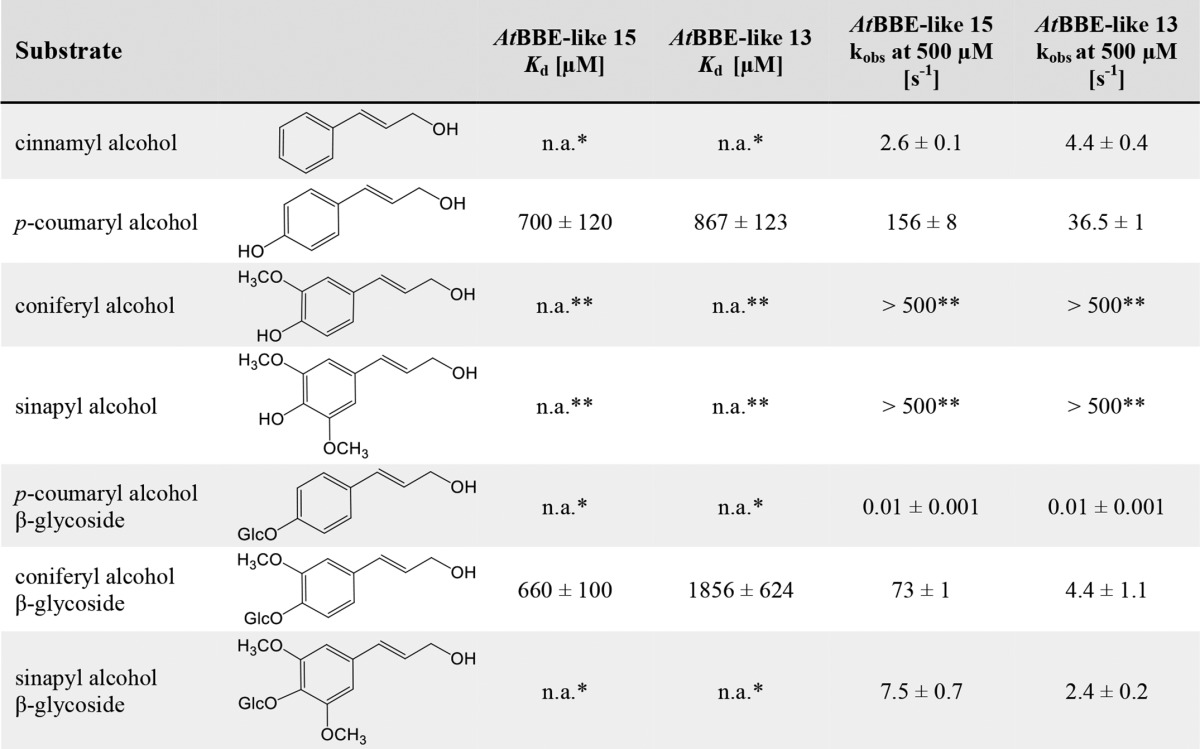
**Chemical structures of substrates and their kinetic parameters for *At*BBE-like 13 and *At*BBE-like 15** Dissociation constants and observed rates of reduction of *At*BBE-like 13 and *At*BBE-like 15 with different monolignols and their glycosylated derivatives are shown.

* Data were not determined due to solubility limitation.

** Reaction was too fast to be measured.

Reoxidation of photo-reduced *At*BBE-like 13 and *At*BBE-like 15 by molecular dioxygen was very slow yielding an oxidative rate constant of 3.3 ± 0.6 and 27.5 ± 1.4 m^−1^ s^−1^, respectively, thus indicating that the enzymes suppress the reduction of oxygen by the reduced FAD cofactor. Recently, we have reported that oxygen reactivity in the BBE family is controlled by the side chain of a gatekeeper residue in the “oxygen reactivity loop” on the *re*-side of the isoalloxazine ring. In the case of *At*BBE-like 15, the gatekeeper residue is a leucine (Leu-182), and thus oxygen reactivity is suppressed because the side chain blocks access to the oxygen pocket (38). Replacement of Leu-182 to valine in *At*BBE-like 15 increased the rate of reoxidation ∼400-fold to 1·10^4^ m^−1^ s^−1^, *i.e.* very similar to the rate found with *Ec*BBE (*k*_ox_ = 5·10^4^ m^−1^ s^−1^) (39), which also possesses a valine residue in the gatekeeper position. Because the reaction of the reduced FAD with dioxygen is very slow, we assume that an alternative electron acceptor is required for reoxidation of the reduced FAD cofactor of *At*BBE-like 13 and *At*BBE-like 15 *in planta*.

##### Crystal Structure of AtBBE-like 15

**FIGURE 2.**
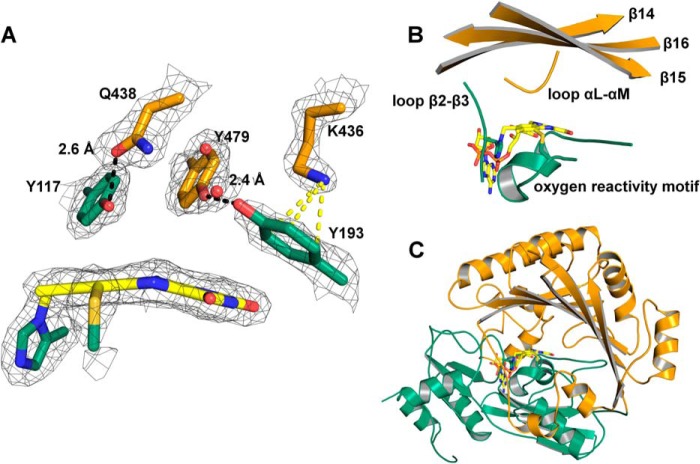
*A,* close-up view of the amino acids with a proposed role in substrate binding and catalysis. Tyr-117 and Gln-438 form a hydrogen bond. Lys-436 and Tyr-193 are involved in a cation-π interaction, and Tyr-193 forms a hydrogen bond to Tyr-479. Two well defined water molecules are found in the active site (*red spheres*). The electron density was cut off at a σ value of 1.5 Å. *B,* close-up view of the active site. Loop β2-β3 and the oxygen reactivity motif originate from the FAD-binding domain and harbor His-115 and Cys-179, which form the two covalent linkages to the isoalloxazine ring of the FAD. Strands β14, β15, and β16 and loop αL-αM originate from the substrate-binding domain. They harbor amino acids defining the *si*-face near the N5 locus (<10 Å) that are putatively involved in the catalytic mechanism. *C,* overall topology of *At*BBE-like 15. *At*BBE-like 15 adopts the VAO topology consisting of a FAD-binding domain (*green*) and a substrate-binding domain (*orange*). Furthermore, it features a bicovalently attached FAD typical for BBE-like proteins. The FAD cofactor is shown in *stick* representation in *yellow*.

To better understand the enzymatic reaction mechanism and substrate binding to the active site, we have elucidated the three-dimensional structure of *At*BBE-like 15 by means of x-ray crystallography (Protein Data Bank code 4UD8). Protein crystals were obtained using sitting drop conditions yielding diffraction data to 2.1 Å. The structure was solved by molecular replacement using *Ec*BBE (Protein Data Bank code 3D2H) with two molecules in the asymmetric unit (data collection and statistics are given in Table 3). *At*BBE-like 15 adopts the same fold as other BBE-like enzymes, *i.e.* the VAO-fold that is characterized by a FAD-binding and a substrate-binding domain (Fig. 2*C*, shown in *green* and *orange*, respectively). The substrate-binding domain consists of a seven-stranded antiparallel β-sheet, which is covered by α-helices. The substrate-binding pocket and the active site are formed by two short loops (termed loop β2-β3 and loop αL-αM), the “oxygen reactivity motif” consisting of loop αD-αE, helix αE, and loop αE-β6. Additionally, the three strands β14, β15, and β16 contribute to the active site (Fig. 2*B*, nomenclature of secondary structure elements according to Ref. 15). The FAD cofactor is bicovalently linked to the peptide chain via a covalent bond of His-115 to the 8α-methyl group and of Cys-179 to the C6-position of the isoalloxazine ring.

**TABLE 3:** **Data acquisition and refinement parameters for x-ray crystallography**

	*At*BBE-like 15
Beamline	Elettra XRD1
Wavelength (Å)	0.971670
Unit cell parameters (Å,°)	63.6, 94.7, 188.3, 90, 90, 90
Space group	*P*2_1_2_1_2_1_
Resolution limits (Å)	50-2.09 (2.21-2.09)
*R*_meas_	0.209 (0.865)
*R*_merge_	0.191 (0.794)
Total no. of observations	415,743 (58,987)
Total no. unique	67,102 (9501)
〈*I*/σ(*I*)〉	7.73 (2.23)
Completeness (%)	97.8 (87.0)
Redundancy	6.2
Wilson *B* factor (Å^2^)	32.02
Matthews coefficient (Å^3^ Da^−1^)	2.36
Molecules per ASU	2
Solvent content (%)	48

**Refinement**	
Resolution (Å)	47.37-2.09
R (%)	18.35
*R*_free_ (%)	22.09

**Root mean square deviation stereochemistry**	
Bond lengths (Å)	0.008
Bond angles (°)	0.825
No. of protein atoms	7946
No. of non-protein atoms	247
No. of water molecules	615
Average -*B-*factor (Å^2^)	25.54

**Ramachandran analysis**	
Favored (%)	95.96
Allowed (%)	3.94
Disallowed (%)	0.1
PDB code	4ud8

As hydride transfer from the substrate to the N5 locus of the flavin cofactor is a generally accepted mode of reduction, a sphere with a radius of 10 Å around this locus was examined for putative catalytic residues. As shown in Fig. 2*A*, Tyr-117 and Gln-438 are engaged in a hydrogen bond on the *si*-side of the isoalloxazine ring (2.6 Å). These residues are positioned between the active site and the binding pocket and thus must interact with the substrate when it enters the active site. Therefore, these residues were defined as substrate coordination motif. The hydroxyl groups of Tyr-117, Tyr-479, and Tyr-193 point toward the active site with distances to the N5 locus of 4.8, 4.6, and 5.0 Å, respectively. The two phenolic hydroxyl groups of Tyr-479 and Tyr-193 are within hydrogen bond distance (2.4 Å, Fig. 2*A*). The ϵ-amino group of Lys-436 is located 3.1 Å above the plane of the aromatic ring of Tyr-193 and engages in a cation-π interaction. This arrangement of Tyr-479, Tyr-193, and Lys-436 presumably favors deprotonation of the phenolic hydroxyl group of Tyr-193 and activates this residue as a putative active site base, and thus these residues were defined as a catalytic base motif.

##### Docking of Substrates to the Active Site

**FIGURE 3.**
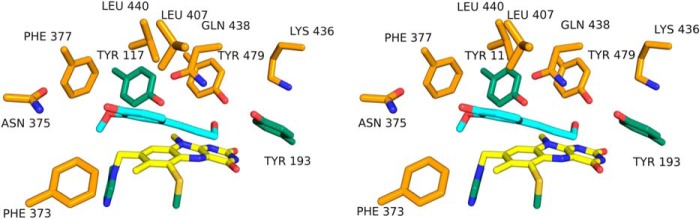
**Substrate binding to *At*BBE-like 15.** The substrate-binding cavity and active site of *At*BBE-like 15 are shown in stereo view. The isoalloxazine ring is shown in *yellow* and coniferyl alcohol in *light blue* as a *stick* model. Coniferyl alcohol was docked into the cavity using YASARA. The aromatic moiety is located in the hydrophobic binding pocket formed by Phe-377, Phe-373, Leu-407, Leu-440, and Tyr-117, and the isoalloxazine ring, whereas the allyl alcohol is facing the active site. All residues shown in this figure are conserved in *At*BBE-like 13.

Because co-crystallization with the identified substrates was unsuccessful, we conducted docking experiments using coniferyl alcohol as a model substrate to gain further insights into the interaction of substrates with amino acid residues in the active site. At the *si*-side of the isoalloxazine ring, a hydrophobic pocket is formed by residues Phe-373, Phe-377, Leu-407, and Leu-440 and the dimethylbenzene moiety of the isoalloxazine ring. As shown in Fig. 3, the substrate fits into this hydrophobic pocket (binding energy of −6.4 kJ mol^−1^). The docking result suggests that the orientation of the allylic alcohol toward the proposed catalytic base Tyr-193 is possible, although the aromatic ring is located in the hydrophobic pocket, and thus this orientation is consistent with the productive catalysis found for *At*BBE-like 15 with this ligand.

##### Functional and Structural Characterization of the BBE-like Family in A. thaliana

**FIGURE 4.**
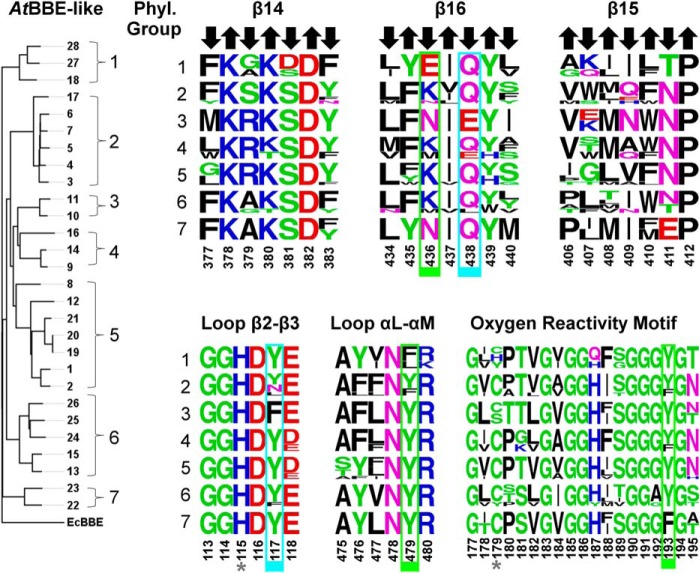
**Chemical nature of the active sites present in different phylogenetic groups is represented by sequence logos of the active site forming secondary structure elements.** *Left,* protein distance tree of all BBE-like enzymes from *A. thaliana* with *Ec*BBE as outgroup. *Right,* sequence logos representing the structural elements forming the active site of the seven phylogenetic groups reveal highly conserved motifs. *Arrows* indicate the orientation of the residues in the β-strands. An *upward arrow* indicates that the residue points away from the active site. These residues were found to be structurally relevant. A *downward arrow* indicates the residue points toward the active site and thereby contributes to the decoration. Residues highlighted in a *green* and *cyan boxes* define the substrate coordination and catalytic motifs, respectively. Both motifs are putatively involved in the catalytic mechanism (see also Fig. 5 and 6). Positions labeled with an *asterisk* highlight the two potential sites of covalent linkage.

The identification of critical active site residues with a role in substrate binding and catalysis in *At*BBE-like 15 raised the question whether these are conserved in all *At*BBE-like family members. To investigate this issue, we have constructed a protein distance tree (Fig. 4, *left*). Seven distinct groups can be identified in the phylogenetic tree within the family of BBE-like enzymes in *A. thaliana*, in good agreement with previously published work (9). Based on these distinct groups, we have created sequence logos for each of the elements forming the active site, *i.e.* the three strands at the ceiling of the active site cavity and the three loops in the vicinity of the isoalloxazine ring (Fig. 4, *right*). The histidine residue (His-115) in loop β2-β3 that forms the covalent bond to the 8α-methyl group of the flavin isoalloxazine ring is conserved in all *At*BBE-like enzymes, and thus it can be assumed that the covalent linkage is present in all family members. In contrast, the cysteine residue (Cys-179) located in the oxygen reactivity motif is only conserved in phylogenetic groups 2, 4, 5 and 7, whereas the remaining groups feature other amino acids, such as histidine (group 1), serine (group 3), or tyrosine (group 1 and 6). We assume that these four *At*BBE-like enzymes possess a single covalent linkage, whereas the remaining 24 feature a bicovalent linkage of the FAD cofactor as already shown for *Ec*BBE (40), Dbv29 (41), glucooligosaccharide oxidase (GOOX) (42), and THCA synthase (15).

**FIGURE 5.**
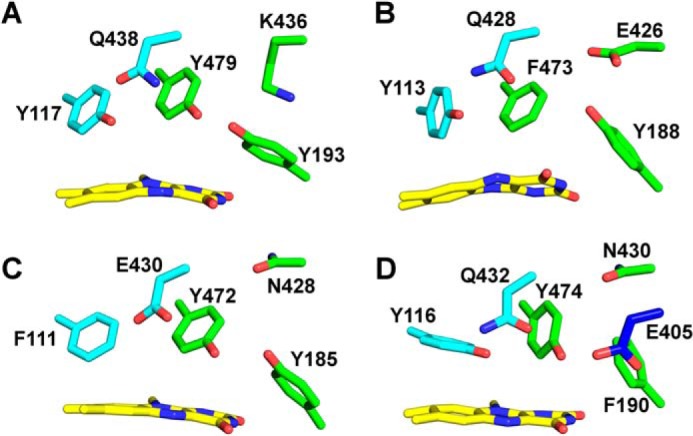
**Active site composition predominantly present in different phylogenetic groups.** The active site compositions shown in *B, C,* and *D* were visualized using homology models created with YASARA. *A,* active site composition of *At*BBE-like 15 that is representative for groups 2 and 4–6. *B,* this active site composition is found in group 1 (numbering according to *At*BBE28). The catalytic base as found in *At*BBE-like 15 has been disrupted. Lys-436 has been replaced by a glutamic acid that putatively can recover the catalytic base function. *C,* this active site composition is found in group 3 (numbering according to *At*BBE11). The catalytic base motif is inconsistent; Gln-438 has been replaced by a glutamic acid that putatively recovers the catalytic base function. *D,* active site composition as found in group 7 (numbering according to *At*BBE22). In the catalytic base, motif Lys-436 has been replaced by an asparagine and Tyr-193 by a phenylalanine. In β15, a glutamic acid can be found that putatively recovers the catalytic base function in this active site.

Further analysis of the sequence logos generated for the three strands of the β-sheet provided important insights into the functions of amino acid side chains. Generally, amino acids oriented toward the α-helices (Fig. 4, *upward pointing arrows*) are conserved because they are important for the structural integrity of the protein. For example, Lys-380 and Asp-382 in strand β14 form salt bridges to Asp-481 (2.7 Å) and Arg-473 (3.3 Å), respectively. Thus, these amino acids are invariant in the whole family, because all of them adopt the same overall topology. In contrast, amino acid residues pointing toward the active site (Fig. 4, *downward pointing arrows*) are only conserved within certain phylogenetic groups, indicating that the composition of the active site differs markedly. In the case of *At*BBE-like 15 (group 6 in Fig. 4), the active site residues Tyr-117, Tyr-193, Lys-436, Gln-438, and Tyr-479 are important for substrate binding and catalysis (*boxed residues* in Fig. 4 and shown as *stick models* in Fig. 5*A*). While residues Tyr-193, Tyr-479, and Lys-436 concertedly act as an active site base (Fig. 5*A,* “catalytic motif,” shown in *green*), Tyr-117 and Gln-438 appear to be involved in determining the substrate preference (“substrate coordination motif,” shown in *cyan*). Both motifs are strictly conserved in 14 members of the BBE-like enzymes in *A. thaliana* (see Table 4), indicating that their catalytic mechanism and substrate preference will be similar, *i.e.* oxidation of alcohols to their corresponding aldehyde products. This analysis was extended to other plants revealing that the active site signature found in *At*BBE-like 13 and *At*BBE-like 15 is conserved in soybean (*Glycine max*; 21 homologs of 43 BBE-like enzymes), eucalyptus (*Eucalyptus grandis*; 18 of 27), poplar (*P. trichocarpa*; 17 of 64), and potato (*Solanum tuberosum*; 6 of 18). Interestingly, monocotyledonous species such as maize (*Zea mays*; 16 BBE-like enzymes) and rice (*Oriza sativa*, 11 BBE-like enzymes) appear to lack monolignol oxidoreductase activity, because the catalytic and substrate coordination motifs are not found in any of the BBE-like enzymes.

**TABLE 4:**
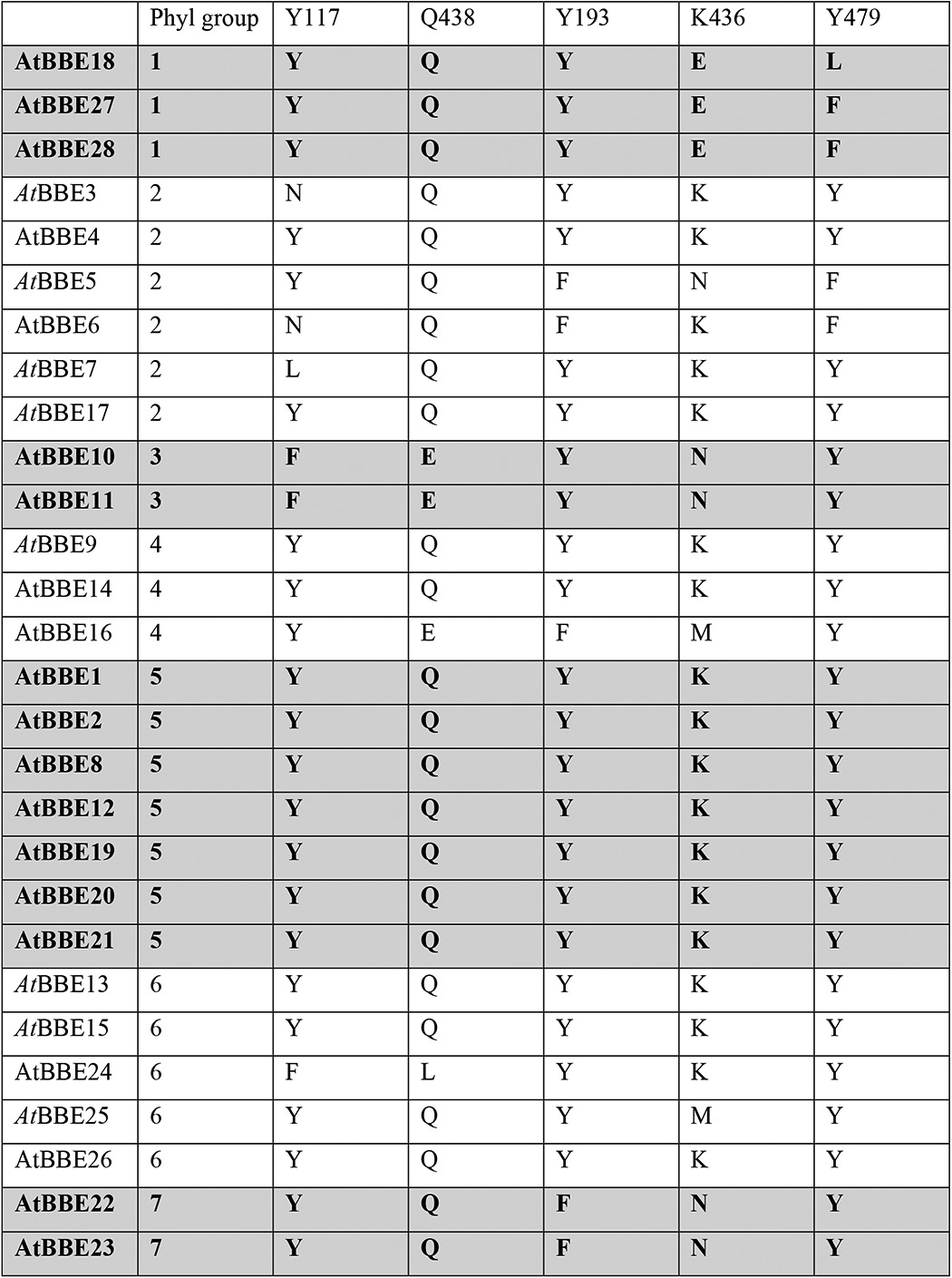
**Composition of the substrate coordination (Tyr-117 and Gln-438) and catalytic motif (Tyr-193, Lys-436, and Tyr-479)**

In contrast, major variations in the catalytic and/or substrate coordination motif are seen in groups one, three, and seven. In group one, Lys-436 in the catalytic motif is replaced by glutamic acid (Fig. 4, strand β16, and Fig. 5*B*) and Tyr-479 by phenylalanine or leucine, respectively (Fig. 4, α*L*-α*M*), although the substrate coordination motif is conserved. In group three, the substrate coordination motif (Tyr-117 and Gln-438 are replaced by phenylalanine and glutamic acid, respectively) as well as the catalytic motif residues (Lys-438 replaced by asparagine) are modified (Fig. 5*C*). Group seven features a conserved substrate coordination motif, but in the catalytic motif residues Lys-436 and Tyr-193 are replaced by asparagine and phenylalanine, respectively (Fig. 5*D*). It is worthwhile noting that in all cases where the catalytic motif appears to be disrupted, an alternative catalytic base is present, for example glutamic acid instead of Lys-436 or Asn-411 in groups one and seven, respectively. Similarly, Gln-438 in group three is replaced by glutamic acid. Overall, we conclude that the BBE-like enzymes of these groups have distinct catalytic properties and act on different yet unidentified substrates. Interestingly, the presence of a catalytic active base appears to be an important feature in BBE-like enzymes, as it was also observed in *Ec*BBE (16), Dbv29 (41), GilR (43), GOOX (42), and THCA synthase (15).

## Discussion

### 

#### 

##### Catalytic Mechanism of Monolignol Oxidation

**FIGURE 6.**
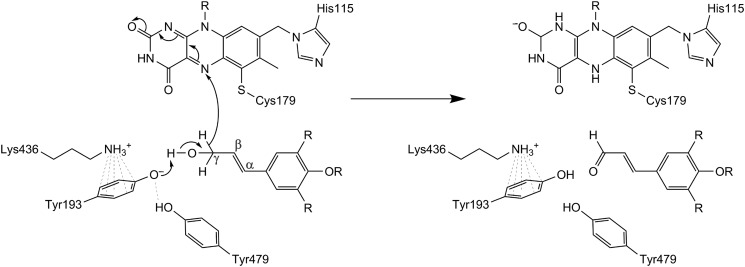
**Proposed reaction mechanism for substrate oxidation.** Tyr-193, Tyr-479, and Lys-436 concertedly act as an active site base abstracting a proton from the alcohol with subsequent hydride transfer to the N5 of the isoalloxazine ring.

Based on the crystallographic structure of *At*BBE-like 15 and the results obtained by substrate docking, we propose the following catalytic mechanism. Tyr-193 is positioned such that the phenolic hydroxyl group points toward the substrate's alcohol group. Lys-436 forms a cation-π interaction with the aromatic ring of Tyr-193, thereby lowering the p*K_a_* value and thus stabilizing the deprotonated state. We also assume that Tyr-479 stabilizes the deprotonated state of Tyr-193 by hydrogen bonding (Figs. 2 and 3). Thus, these three amino acids make up the core catalytic machinery for deprotonation of the substrate's hydroxyl group that results in the concomitant transfer of a hydride from the Cγ position to the N5 locus of the isoalloxazine ring of the flavin. A schematic reaction mechanism for the oxidation of monolignols by AtBBE-like 15 is shown in Fig. 6. To test this hypothesis, we have initiated a site-directed mutagenesis study of the pertinent active site residues.

##### Structural Comparison with Other Members of the VAO Family

**FIGURE 7.**
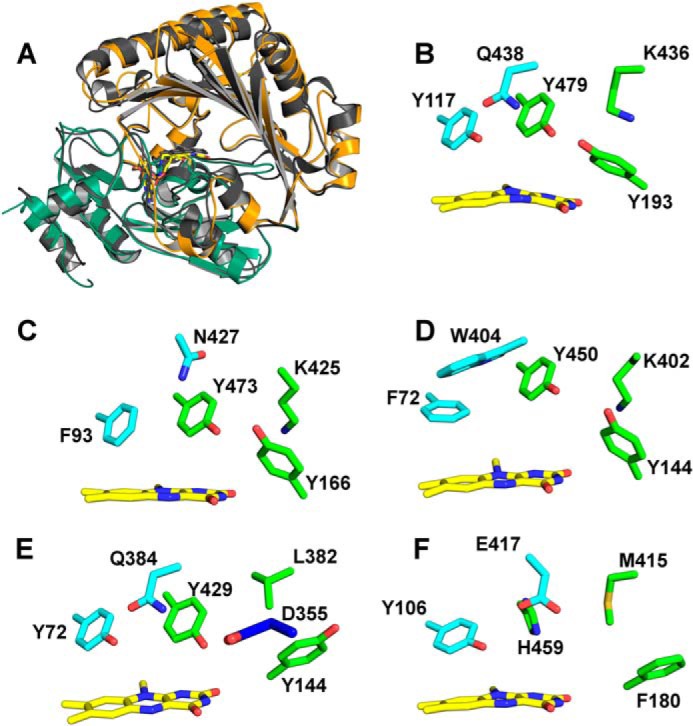
**Comparison of overall topology and active site composition of various BBE-like enzymes and VAO superfamily members.** *A,* overall topology of *At*BBE-like 15 and superposition with *Ec*BBE. *B–F* show the substrate coordination and catalytic motif in *cyan* and *green*, respectively, for the following BBE-like proteins. *B, At*BBE-like 15, the substrate coordination motif, consists of Tyr-117 and Gln-438, and the catalytic motif is formed by Tyr-479, Tyr-193, and Lys-436. *C,* Dbv29, the catalytic motif is invariant, whereas the substrate coordination motif is different. *D,* AknOx, the catalytic motif is invariant, whereas the substrate coordination motif differs. *E,* GOOX, the catalytic motif is disrupted, and it appears to be restored by the presence of another catalytic base, *i.e.* Asp-355. *F, Ec*BBE, both motifs are disrupted with Glu-417 serving as a catalytic base.

In recent years, the structures of several other members of the VAO family were determined that share a high degree of structural similarity despite low similarity on the sequence level. Structural superposition of *At*BBE-like 15 with Dbv29, GOOX, AknOx, and *Ec*BBE gives a root mean square deviation of 1.45 Å (for 1652 backbone atoms), 1.36 Å (for 1556 backbone atoms), 1.47 Å (for 1526 backbone atoms), and 0.95 Å (for 2068 backbone atoms) respectively, whereas sequence identities of 22, 22, 23, and 41%, respectively, are found. The bacterial oxidases Dbv29 and AknOx catalyze similar oxidations of alcohol groups and thus possess a very similar catalytic motif consisting of both tyrosine residues and the lysine residue also present in *At*BBE-like 15, whereas the substrate recognition motif is clearly different as depicted in Fig. 7 (*C* and *D*, respectively). In any case, these enzymes act on alcohol substrates that show no similarity to the substrates identified for *At*BBE-like 15 and 13 and, moreover, are not related to lignin metabolism. Importantly, for Dbv29 and AknOx it was found that both tyrosine residues are crucial for catalysis thus supporting the proposed role of Tyr-193 and Tyr-479 in the mechanism shown in Fig. 6 for *At*BBE-like 15 (and *At*BBE-like 13) (10, 41). Although Dbv29 and AknOx resemble *At*BBE-like 15 and 13 concerning the composition of the active site on the *si*-side of the isoalloxazine ring, the residue that determines the reactivity toward dioxygen, *i.e.* Leu-182 in the case of *At*BBE-like 15, is a valine in both enzymes, defining them as oxidases (38). The active site of GOOX has the same substrate recognition motif but has a distinct catalytic motif featuring a glutamate residue (shown in *blue* in Fig. 7*E*) instead of the tyrosine and lysine seen in *At*BBE-like 15 (compare *B* and *E* in Fig. 7). In fact, this active site composition is similar to group one of the *At*BBE-like family, and thus it is conceivable that these BBE homologs are also involved in the oxidation of carbohydrates as already reported for some plant BBE-like enzymes (8, 9). Not surprisingly, the biggest difference in active site composition is seen with *Ec*BBE as this enzyme catalyzes an unusual oxidative ring closure reaction in alkaloid biosynthesis; although the overall topology shows high similarity (Fig. 7*A*), except for the tyrosine in the substrate recognition motif (Tyr-106), all other residues vary (compare *B* and *F* in Fig. 7). Moreover, Glu-417 replacing the glutamine of the substrate recognition motif assumes a crucial role in the reaction mechanism of *Ec*BBE and thus becomes part of the catalytic machinery of the enzyme (16). This comparison illustrates that this enzyme family can be tuned for new reactivities by strategic single or multiple exchanges of amino acids in the active site. In fact, even within a given active site different (oxidation), reactions are supported depending on the available substrates further demonstrating the plasticity of the active site (44).

##### Proposed Role of AtBBE-like 15/Monolignol Oxidoreductases in Plants

Monolignols are secreted into the apoplast and polymerized to various cell wall components. Although monolignols are thought to be secreted in their alcohol forms, not all monolignol-derived cell wall components are coupling products of monolignols but the aldehydes are also incorporated into the cell wall (45). It has been shown by mass spectrometry that AtBBE-like 15 is located in the apoplastic fluid of *A. thaliana,* and thus our findings that AtBBE-like 15 (and *At*BBE-like 13) oxidizes monolignols to their corresponding aldehydes indicates that they are likely physiological substrates (46). In addition, the glycosylated form of coniferyl alcohol (coniferin) is also accepted as substrate. Additionally, oxidation of coniferin to its corresponding aldehyde has been postulated by Tsuji *et al.* (47) for the plant *Gingko biloba*. According to their hypothesis, oxidation of the β-glycosylated monolignols precedes hydrolysis to the free monolignols and thus plays an important role for the mobilization of monolignols from their glycosidic storage forms. Hence, the demonstrated activity of *At*BBE-like 13 and *At*BBE-like 15 *in vitro* suggests that the enzymes may participate in the mobilization and oxidation of monolignols required for polymerization processes in the plant cell wall (*e.g.* lignification). Interestingly, more than 20 different glycoside hydrolases were also identified during this proteome analysis, suggesting that *At*BBE-like 15 and potentially also other members of the family work in concert with these hydrolases to mobilize building blocks from their storage forms. This concept receives further support by co-expression data retrieved from the ATTET II server (CoExSearch Version 4.1). Among the enzymes co-expressed with *At*BBE-like 15 are phenylalanine ammonia-lyase 4 (PAL4), an important rate-determining entry point of the phenylpropanoid pathway, and β-glucosidase 41 clustering with the monolignol glucoside hydrolases At1g61810 (BGLU45) and At1g61820 (BGLU46) (see supplemental Table S2) (48, 49). Thus, we suggest that *At*BBE-like 13 and *At*BBE-like 15 participate in the adaptation of the extracellular monolignol pool prior to polymerization and are thus involved in cell wall formation required during certain growth phases and in response to stressors. Because the active site motifs found in *At*BBE-like 13 and *At*BBE-like 15 are conserved in 12 other members of the BBE-like enzyme family in *A. thaliana,* we predict that they play similar roles in the plant potentially with diverging substrate specificities or, alternatively, with different spatial (*i.e.* different tissues) or temporal (*i.e.* induced by different biotic or abiotic stressors) features. The presence of homologs of *At*BBE-like 13 and *At*BBE-like 15 in many other plants (see above) indicates a general role of this enzyme family in the plant kingdom. In contrast, enzymes such as *Ec*BBE and THCA synthase serve in “secondary” pathways restricted to certain plant families or genera, and hence these have evolved, *e.g.* by gene duplication and subsequent diversification by mutation, from the primordial monolignol oxidoreductases, a phenomenon well documented for the evolution of new plant defense compounds (50).

From the analysis of the active site signatures, it is also evident that *At*BBE-like enzymes in phylogenetic groups one, three, and seven possess distinct catalytic machineries and probably act on as yet unidentified substrates. These BBE homologs are currently the target of further studies to reveal their biochemical properties and physiological function. In addition, we have initiated the generation, identification, and characterization of gene knock-out plants with the aim to investigate the role of *At*BBE-likes in cell wall metabolism during plant development and stress response.

## Author Contributions

B. D., K. G., and P. M. initiated and designed the research; B. D., B. S., and S. W. carried out biochemical research; A. G. and B. N. synthesized glycosylated monolignol derivatives; A. D., T. P.-K., B. D., and K. G. crystallized proteins and solved the x-ray crystal structure; B. D., C. S., and E. v. d. G. performed phylogenetic analysis and evaluated the data; B. D., E. v. d. G., K. G., and P. M. wrote the manuscript.

## Supplementary Material

Supplemental Data

## Footnotes

2 The abbreviations used are:

BBE: berberine bridge enzyme
THCA: Δ1-tetrahydrocannabinolic acid synthase
VAO: vanillyl alcohol oxidase
GOOX: glucooligosaccharide oxidase.
