# Supplementary material for: Oxidation of Monolignols by Members of the Berberine Bridge Enzyme Family Suggests a Role in Plant Cell Wall Metabolism
Source: J Biol Chem. 2015 Jun 2;290(30):18770–81. doi: 10.1074/jbc.M115.659631 (PMC4513132; doi:10.1074/jbc.M115.659631)
Supplement: Supplemental Data [file supp_290_30_18770__index.html]

Oxidation of Monolignols by Members of the Berberine Bridge Enzyme Family Suggests a Role in Cell Wall Metabolism — Oxidation of Monolignols by Members of the Berberine Bridge Enzyme Family Suggests a Role in Plant Cell Wall Metabolism — Oxidation of Monolignols by BBE-like Enzymes — Supplemental Data 

# Oxidation of Monolignols by Members of the Berberine Bridge Enzyme Family Suggests a Role in Plant Cell Wall Metabolism

## Supplemental Data

- supplementat information (.pdf, 808 KB) - allowable supplemental information
