# Supplementary material for: Oxidation of Monolignols by Members of the Berberine Bridge Enzyme Family Suggests a Role in Plant Cell Wall Metabolism
Source: J Biol Chem. 2015 Jun 2;290(30):18770–81. doi: 10.1074/jbc.M115.659631 (PMC4513132; doi:10.1074/jbc.M115.659631)
Supplement: Supplemental Data [file supp_M115.659631_jbc.M115.659631-1.pdf]

Oxidation of Monolignols by Members of the Berberine Bridge Enzyme Family Suggests a Role in Plant Cell Wall Metabolism

Supplementary Information

Bastian Daniel,[1] Tea Pavkov-Keller[2,3], Barbara Steiner,[1] Anđela Dordic[2,3], Alexander Gutmann,[4] Bernd Nidetzky,[4] Christoph W. Sensen,[5] Eric van der Graaff,[6] Silvia Wallner,[1] Karl Gruber[2] and Peter Macheroux[1*†]

[1]Graz University of Technology, Institute of Biochemistry, Graz, Austria

[2]University of Graz, Institute of Molecular Biosciences, Graz, Austria

[3]Austrian Centre of Industrial Biotechnology, Graz, Austria

[4]Graz University of Technology, Institute of Biotechnology and Biochemical Engineering, Graz, Austria

[5]Graz University of Technology, Insitute of Molecular Biotechnology, Graz, Austria

[6]Copenhagen University, Copenhagen, Denmark

Table S1: Thermofluor measurements with selected compounds

Substance	CAS-number	T_m [°C]	ΔT_m [°C]
2-Deoxyglucose	154-17-6	78	17
D,L-Proline	609-36-9	77.5	16.5
trans-Cinnamyl alcohol	104-54-1	76.5	15.5
D-Glucose	492-62-6	76	15
3-Aminobenzyl alcohol	1877-77-6	75	14
3,4-Dimethoxybenzyl alcohol	93-03-8	75	14
4-Isopropylbenzyl alcohol	536-60-7	74.5	13.5
(*S*)-(-)-Limonen	5989-54-8	74	13
Benzylalcohol	100-51-6	73	12
Geraniol	106-24-1	73	12
Lactose monohydrate	64044-51-5	72	11
D-Maltose		72	11
Anthranilic acid	118-92-3	71	10
Cellobiose	528-50-7	71	10
4-Chlorobenzyl alcohol	873-76-7	71	10
D-Galactose	59-23-4	71	10
D-Sorbitol	50-70-4	71	10
Furfuryl alcohol	98-00-0	70.5	9.5
2-Indanol	4254-29-9	70.5	9.5
Polygalacturonic acid	25990-10-7	70	9
(+)-Pulegone	89-82-7	70	9
Eugenol	97-53-0	65.5	4.5
Xylose	58-86-6	66.5	5.5
3-Methoxyphenol	150-19-6	64.5	3.5
D-Lyxose	1114-34-7	64	3
Acetylacetone	123-54-6	63.5	2.5
3-(3,4-Dihydroxyphenyl)-DL-alanine (L-DOPA)	63-84-3	63.5	2.5
3-Aminosalicyl acid	570-23-0	63	2
2-Aminothiophenol	137-07-5	63	2
p-Anisidine	104-94-9	63	2
(*S*)-(-)-ß-Citronellol	7540-51-4	63	2
3,4-Dimethoxybenzonitrile	2024-82-1	63	2
Flavone	525-82-6	63	2
Isopropyl salicylate	607-85-2	63	2
Vanillin	121-33-5	63	2
meso-Erythritol	149-32-6	62.5	1.5
2-Acetyl-5-bromothiophene	5370-25-2	62	1
1,1'-Bi-2-naphthol	602-09-5	62	1
3-Chlorocoumarin	92-45-5	62	1
Dihydrocarveol	619-01-2	62	1
3-Aminophenol	591-27-5	61.5	0.5
Coumarin	91-64-5	61.5	0.5
Diacetyldioxim	95-45-4	61.5	0.5
2,6-Lutidin	108-48-5	61.5	0.5

(+/-)-Mandelic acid	611-72-3	61.5	0.5
Acetone cyanohydrin	75-86-5	61	0
Acetonylacetone	110-13-4	61	0
Benzamide	55-21-0	61	0
(*R*)-(-)-3-Chloromandelic acid	61008-98-8	61	0
trans-Cinnamonitrile	1885-38-7	61	0
o-Cresol	95-48-7	61	0
1,2-Dihydronaphtalen	446-53-0	61	0
4,4'-Dimethoxybenzoin	119-25-8	61	0
2,6-Dimethoxyphenol	91-10-1	61	0
2,6-Dimethylaniline	87-62-7	61	0
2,6-Dimethylanisol	1004-66-6	61	0
8-Hydroxyquinoline	148-24-3	61	0
Maltose	69-79-4	61	0
Mandelonitrile	532-28-5	61	0
D-Mannitol	69-65-8	61	0
exo-Norborneol	497-37-0	61	0
Norcamphor	497-38-1	61	0
Safrole	94-59-7	61	0
Sucrose	57-50-1	61	0
Tannin	1401-55-4	61	0
DL-Tryptophan	54-12-6	61	0
3-Acetylcoumarin	3949-36-8	60.5	-0.5
Adonitol	488-81-3	60.5	-0.5
Amylose	9005-82-7	60.5	-0.5
D-Arabinose	10323-20-3	60.5	-0.5
Benzylamine	100-46-9	60.5	-0.5
p-Bromo-DL-mandelic acid	6940-50-7	60.5	-0.5
rac-Camphor	76-22-2	60.5	-0.5
2-Chlorophenol	95-57-8	60.5	-0.5
Cholin acetate	14586-35-7	60.5	-0.5
4-Chromamone	491-37-2	60.5	-0.5
trans-Cinnamaldehyde	14371-10-9	60.5	-0.5
p-Cresol	106-44-5	60.5	-0.5
L-Frucose	2438-80-4	60.5	-0.5
Galactan	9037-55-2	60.5	-0.5
Hydroxyethylcellulose	9004-62-0	60.5	-0.5
Isobutyl cinnamate predominantly trans	122-67-8	60.5	-0.5
cis-Jasmone	488-10-8	60.5	-0.5
Lactulose	4618-18-2	60.5	-0.5
Methyl-D-mannopyranoside	617-04-9	60.5	-0.5
3-Acetylpyrridine	350-03-8	60	-1
Adenine	73-24-5	60	-1
Aniline	62-53-3	60	-1
Anisole	100-66-3	60	-1
Anthranilic acid	118-92-3	60	-1
Arabuigalactan	9036-66-2	60	-1

Butyrophenone	495-40-9	60	-1
Carboxymethylcellulose	9004-32-4	60	-1
Carrageen		60	-1
Carvacrol	499-75-2	60	-1
4-Chloro-3-nitrocoumarin	38464-20-9	60	-1
rac-(-)-Citronellic acid	502-47-6	60	-1
Fructose	57-48-7	60	-1
D(+)-Galatosamine hydrochloride	1772-003-8	60	-1
Geranic acid	459-80-3	60	-1
Glucoseamine	3416-24-8	60	-1
Isobutyramide	563-83-7	60	-1
Isophorone	78-59-1	60	-1
4-Isopropyl aniline	99-88-7	60	-1
4-Isopropyl benzoic acid	536-66-3	60	-1
Maltodextrin	9050-36-6	60	-1
Methyl-β-D-glucoside	709-50-2	60	-1
Morpholine	110-91-8	60	-1
2-Naphtol	135-19-3	60	-1
Natriumalginat	9005-38-3	60	-1
D,L-Norvaline	760-78-1	60	-1
8-Quinolinol	148-24-3	60	-1
D-Ribose	50-69-1	60	-1
Trehalose Dihydrat	6138-23-4	60	-1
2,3,3-Trimethylindolenine	1640-39-7	60	-1
Xylitol	87-99-0	60	-1
L-Xylose	609-06-3	60	-1
m-Anisaldehyde	591-31-1	59.5	-1.5
m-Cresol	108-39-4	59.5	-1.5
Indole	120-72-9	59.5	-1.5
Methylnicotinate	93-60-7	59.5	-1.5
Methylvaleraldehyde	123-15-9	59.5	-1.5
L-Arabinose	5328-37-0	59	-2
Cellulose	9004-34-6	59	-2
Chitin	1398-61-4	59	-2
4-Chlorophenol	106-48-9	59	-2
D-Gluconic acid	526-95-4	59	-2
Pyrocatechol	120-80-9	59	-2
Quinoline	91-22-5	59	-2
Xylan	9014-63-5	59	-2
D,L-Phenylalanine	150-30-1	58.5	-2.5
Glucuronolactone	32449-92-6	58	-3
Mannan	9036-88-8	58	-3
D(+)-Glucuronic acid monohydrate	207300-70-7	56.5	-4.5
Valeraldehyde	110-62-3	56	-5
(+/-)-Menthol	89-78-1	55	-6
(-)-Ephedrine	299-42-3	54	-7
Gallic acid	149-91-7	54	-7

Benzoic acid	65-85-0	53.5	-7.5
4-Aminobenzoic acid	150-13-0	50	-11
3-Methylbutylaldehyde (Isovaleraldehyde)	590-86-3	48	-13
3,5-Dihydroxybenzoic acid	99-10-5	40.5	-20.5
D-Mandelic acid	611-71-2	37	-24
4-Acetylbenzoic acid	586-89-0	---	---
2-Acetyl-1-methylpyrrole	932-16-1	---	---
2-Aminophenol	95-55-6	---	---
4-Aminophenol	123-30-8	---	---
5-Aminosalicylic acid	89-57-6	---	---
trans-Anethole	4180-23-8	---	---
p-Anisaldehyde	123-11-5	---	---
p-Anisic acid	100-09-4	---	---
L-Arabinose	5328-37-0	---	---
Benzaldehyde	100-52-7	---	---
Caffeic acid	331-39-5	---	---
3-Chlorobenzylamine	4152-90-3	---	---
3-Chloro-2-norboranone	30860-22-1	---	---
trans-Cinnamic acid	140-10-3	---	---
Curcumin	458-37-7	---	---
2,4-Dihydroxybenzoic acid	89-86-1	---	---
2,5-Dihydroxybenzoic acid	490-79-9	---	---
4-Dimethylaminopyridine, DMAP	1122-58-3	---	---
4-Ethylaniline	589-16-2	---	---
Hydrocinnamaldehyde	104-53-0	---	---
4-Hydroxy-3-nitrocoumarin	20261-31-8	---	---
2-Iodobenzoic acid	88-67-5	---	---
cis/trans Isoeugenol	97-54-1	---	---
Kojic acid	501-30-4	---	---
Lignin		---	---
L-Mandelic acid	17199-29-0	---	---
1-Napthol	90-15-3	---	---
Pectin	9000-69-5	---	---
Pyrogallol	87-66-1	---	---
Salicylaldehyde	90-02-8	---	---
Valeric acid	109-52-4	---	---

For details of the experimental set-up see “Materials & Methods”;

---, in the presence of these compounds no reproducible signal was obtained;

Table S2: Genes coexpressed with *At*BBE-like 15 according to ATTET II server

Rank	Mutual rank	Correlation	Locus	Function
1	2.6	0.72	At1g26820	ribonuclease 3
2	3.5	0.68	At3g10340	phenylalanine ammonia-lyase 4
3	5.3	0.68	At1g62990	KNOTTED-like homeobox of Arabidopsis thaliana 7
4	6.5	0.64	At2g22560	Kinase interacting (KIP1-like) family protein
5	7.3	0.67	At2g14095	
6	7.5	0.67	At1g28470	NAC domain containing protein 10
7	8.1	0.69	At4g16620	nodulin MtN21 /EamA-like transporter family protein
8	8.7	0.68	At4g18425	Protein of unknown function (DUF679)
9	9.5	0.58	At4g24430	Rhamnogalacturonate lyase family protein
10	9.8	0.65	260208_s_at	At1g70670;At1g70680
11	10.9	0.66	At5g04200	metacaspase 9
12	12.4	0.66	At1g11190	bifunctional nuclease i
13	12.4	0.65	At4g04460	Saposin-like aspartyl protease family protein
14	13.0	0.62	At2g47670	Plant invertase/pectin methylesterase inhibitor superfamily protein
15	17.9	0.55	At2g18480	Major facilitator superfamily protein
16	22.4	0.66	267343_at	At2g44260
17	27.8	0.56	At1g23560	Domain of unknown function (DUF220)
18	28.0	0.58	At3g21550	DUF679 domain membrane protein 2
19	28.3	0.60	At5g65530	Protein kinase superfamily protein
20	30.5	0.54	At2g22800	Homeobox-leucine zipper protein family
21	32.2	0.59	At5g58730	pfkB-like carbohydrate kinase family protein
22	33.3	0.63	At4g34320	Protein of unknown function (DUF677)
23	33.6	0.58	At5g54570	beta glucosidase 41
24	34.3	0.62	At5g08480	VQ motif-containing protein
25	35.4	0.53	At3g27200	Cupredoxin superfamily protein

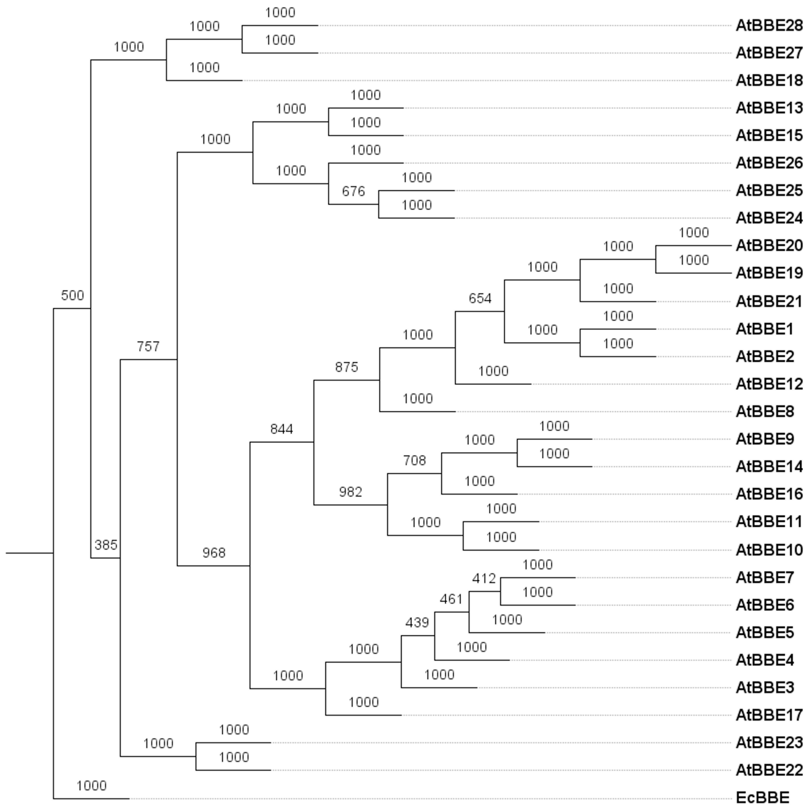

Figure S1: Bootstrapped phylogenetic tree including all *At*BBE-like proteins and *Ec*BBE. The following sequences were used for analysis (locus name/Accession number):
*At*BBE-like 1, AT1G01980.1/Q9LPC3; *At*BBE-like 2, AT1G11770.1/Q9SA99;
*At*BBE-like 3, AT1G26380.1/Q9FZC4; *At*BBE-like 4, AT1G26390.1/Q9FZC5;
*At*BBE-like 5, AT1G26400.1/Q9FZC6; *At*BBE-like 6, AT1G26410.1/Q9FZC7;
*At*BBE-like 7, AT1G26420.1/Q9FZC8; *At*BBE-like 8, AT1G30700.1/Q9SA85;
*At*BBE-like 9, AT1G30710.1/Q9SA86; *At*BBE-like 10, AT1G30720.1/Q9SA87;
*At*BBE-like 11, AT1G30730.1/Q9SA88; *At*BBE-like 12, AT1G30740.1/Q9SA89;
*At*BBE-like 13, AT1G30760.1/Q93ZA3; *At*BBE-like 14, AT1G34575.1/Q9LNL9;
*At*BBE-like 15, AT2G34790.1/O64743; *At*BBE-like 16, AT2G34810.1/O64745;
*At*BBE-like 17, AT4G20800.1/Q9SVG7; *At*BBE-like 18, AT4G20820.1/Q9SVG5;
*At*BBE-like 19, AT4G20830.1/Q9SVG4; *At*BBE-like 20, AT4G20830.2/Q9SVG4;
*At*BBE-like 21, AT4G20840.1/Q9SVG3; *At*BBE-like 22, AT4G20860.1/Q9SUC6;
*At*BBE-like 23, AT5G44360.1/Q9FKV2; *At*BBE-like 24, AT5G44380.1/Q9FKV0;
*At*BBE-like 25, AT5G44390.1/Q9FKU9; *At*BBE-like 26, AT5G44400.1/Q9FKU8;
*At*BBE-like 27, AT5G44410.1/Q9FI25; *At*BBE-like 28, AT5G44440.1/Q9FI21; *Ec*BBE, AAC39358.
